# Delayed Partial Venous Insufficiency of Free Flap: To Intervene or Not?

**DOI:** 10.7759/cureus.51068

**Published:** 2023-12-25

**Authors:** Firoz Borle, Sina Heymans, Simran Dhole, Dushyant Jaiswal

**Affiliations:** 1 Surgery, Jawaharlal Nehru Medical College, Datta Meghe Institute of Higher Education & Research, Wardha, IND; 2 Plastic and Reconstructive Surgery, Marienhospital Stuttgart, Stuttgart, DEU; 3 General Surgery, Jawaharlal Nehru Medical College, Datta Meghe Institute of Higher Education & Research, Wardha, IND; 4 Plastic Surgery, Tata Memorial Hospital, Mumbai, IND

**Keywords:** pectoralis major myocutaneous flap, delayed flap congestion, flap congestion, free flap, head and neck cancer

## Abstract

Delayed venous congestion of a free flap poses a dilemma for clinicians, as the optimal management strategy is often uncertain. This case report presents a successful outcome achieved through a strategy of watchful waiting for a delayed presentation of a partially congested free flap. This approach enabled the avoidance of unnecessary surgical interventions and minimized potential complications associated with flap exploration. By adopting a watchful waiting strategy, clinicians can navigate the challenging decision-making process in cases of partial venous congestion of free flaps, optimizing patient outcomes.

## Introduction

Venous insufficiency of free flaps is the most common indication ( 38%) for re-exploration in the immediate postoperative period [1]. Delayed flap insufficiency (≥5 days postoperatively), arterial, venous, or both, poses a dilemma regarding the appropriate approach for flap salvage. We present a case, where a delayed partially congested free flap was salvaged by watchful waiting [2].

## Case presentation

**Figure 1:**
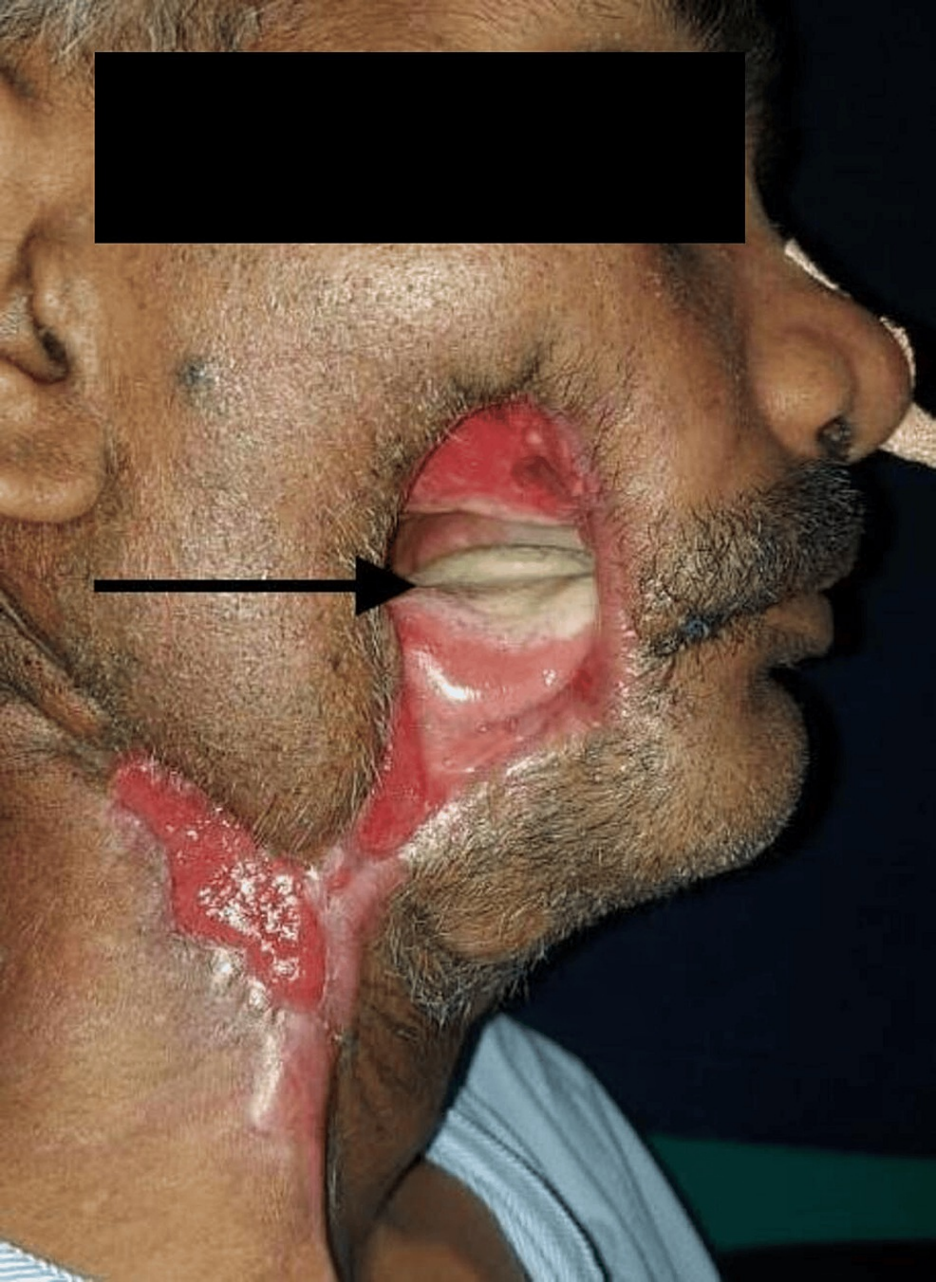
Oro-cutaneous fistula at presentation

A 38-year-old male was diagnosed with squamous cell carcinoma (SCC) of the right buccal mucosa (T4N2BM0). He underwent composite resection of the right buccal mucosa, hemi-mandible, and skin with ipsilateral modified radical neck dissection. Reconstruction was done with a pectoralis major myocutaneous (PMMC) flap for the intraoral lining and a deltopectoral (DP) flap for the skin defect. During the early postoperative period, the DP flap and the skin island of PMMC flaps were necrosed and debrided. Subsequently, the patient had a right carotid blow-out, which was managed by ligating the external carotid artery. Six weeks later the patient presented to our institute with a 7x7 cm oro-cutaneous fistula with granulation over the neck (Figure 1).

**Figure 2:**
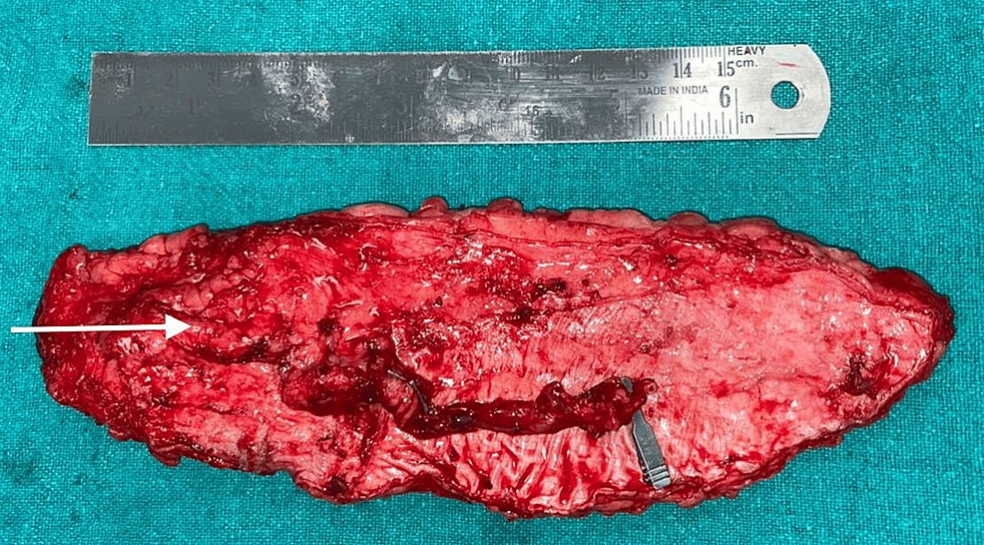
16x7 cm free ALT flap based on single septo-cutaneous perforator ALT, anterolateral thigh

Radiotherapy could not be initiated due to the presence of a fistula. Neither suitable local flap options nor any suitable recipient vessels on the ipsilateral side of the neck for free flap reconstruction were available for closure of the fistula. It was decided to perform closure using a free anterolateral thigh (ALT) flap with anastomosis to contralateral neck vessels using a primary vein graft. A 16x7 cm left ALT flap based on a single septo-cutaneous perforator of the descending branch of the lateral circumflex femoral artery was harvested (Figure 2).

**Figure 3:**
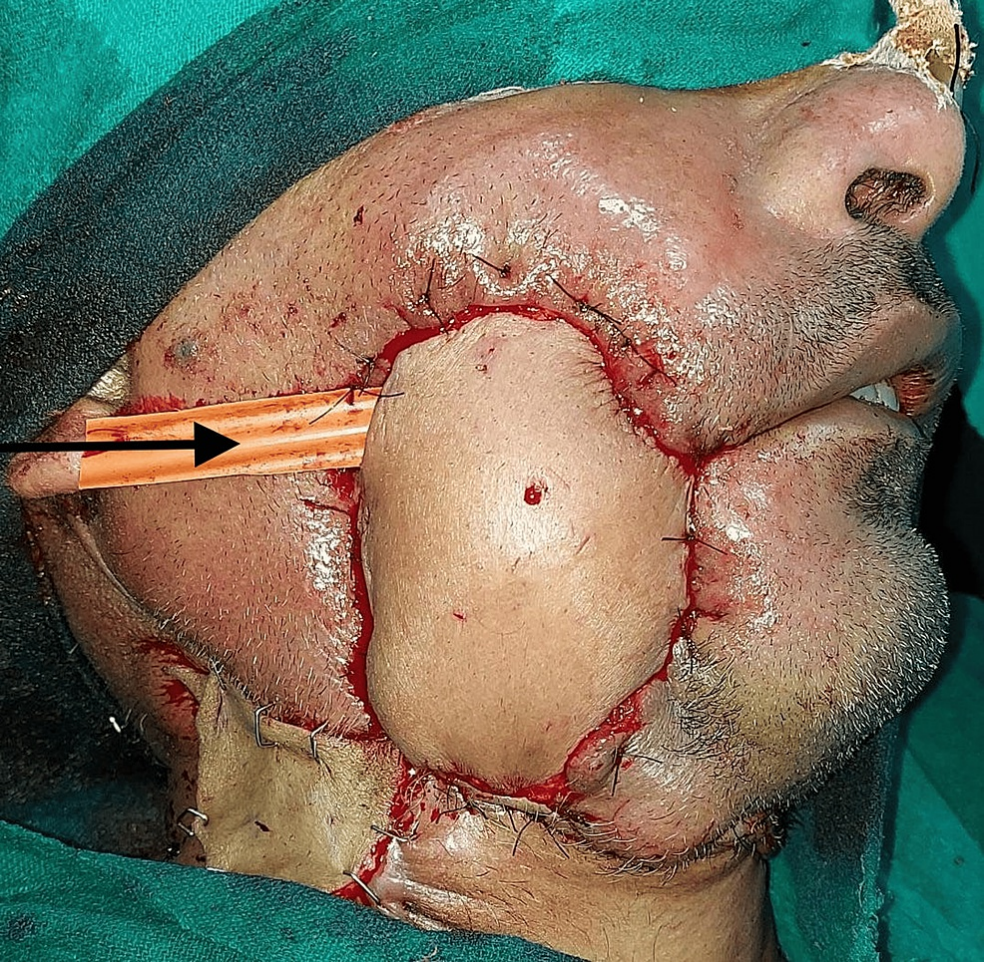
Immediate postoperative picture with arrow depicting drainage through corrugated rubber drain

The flap inset to the buccal mucosa and palate was done. The flap was partially de-epithelialized and brought out to provide skin cover. A great saphenous vein graft was harvested. The flap’s arterial and venous pedicles were anastomosed to the contralateral facial artery and common facial vein, using the vein graft (Figure 3).

**Figure 4:**
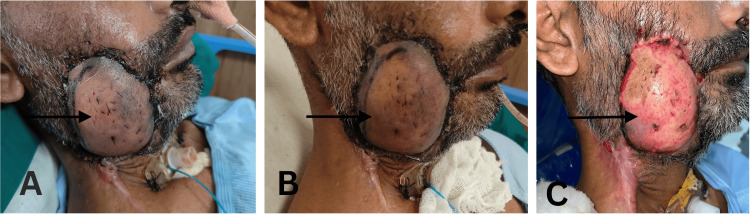
A) Evolution of flap congestion at POD 7. B) Evolution of flap congestion at POD 9. C) Evolution of flap congestion at POD 11 POD, postoperative day

The postoperative course was uneventful until the seventh postoperative day (POD) when the patient had a bout of cough during tracheostomy suctioning. Six hours later the edges of the outer paddle turned dusky, suggestive of venous congestion (Figure 4).

On pinprick, the bleed was dark and brisk at the flap edges, but it was bright red in the 2x3 cm central part of the outer paddle. The inner paddle was normal. On a hand-held Doppler examination, there was a strong signal over the course of the vein graft, suggestive of patent arterial inflow. There were no signs of wound infection. The dilemma was to either explore the microvascular anastomosis (MVA), debridement of the outer skin paddle, and do another flap or watchful waiting. Local/regional pedicle options were exhausted (PMMC/DP was used in the past, and the forehead flap was unviable as ECA was ligated). Another free flap would entail MVA at distant sources, opposite neck again/internal mammary artery/transverse cervical vessels, each requiring vein grafts and a massive surgical effort. 

The decision was taken not to intervene and watchfully wait since the congestion was of the outer part only and the inner paddle was viable. In the following days, POD eighth to 10th, the congestion progressed marginally and by the 11th POD, the progression stopped completely. There was some superficial epidermal necrosis along with marginal necrosis of 2 cm, which was debrided serially. 

**Figure 5:**
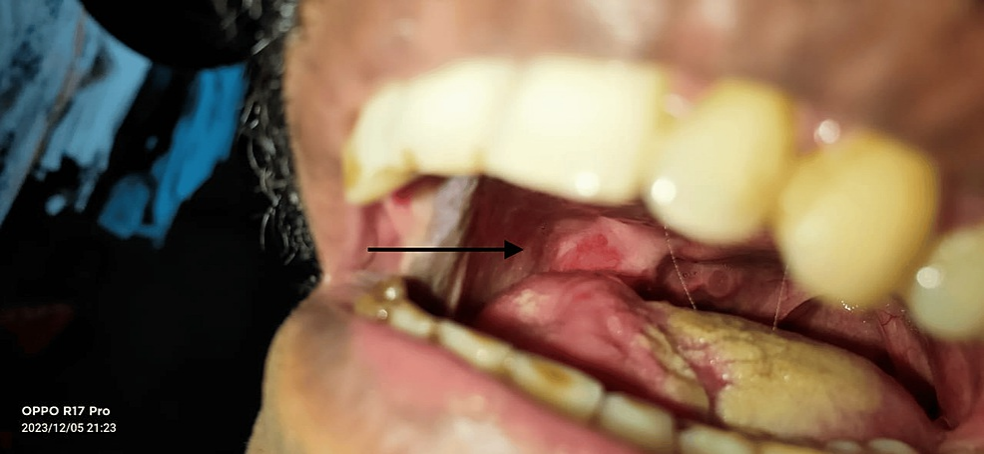
Arrow depicting the intraoral paddle

**Figure 6:**
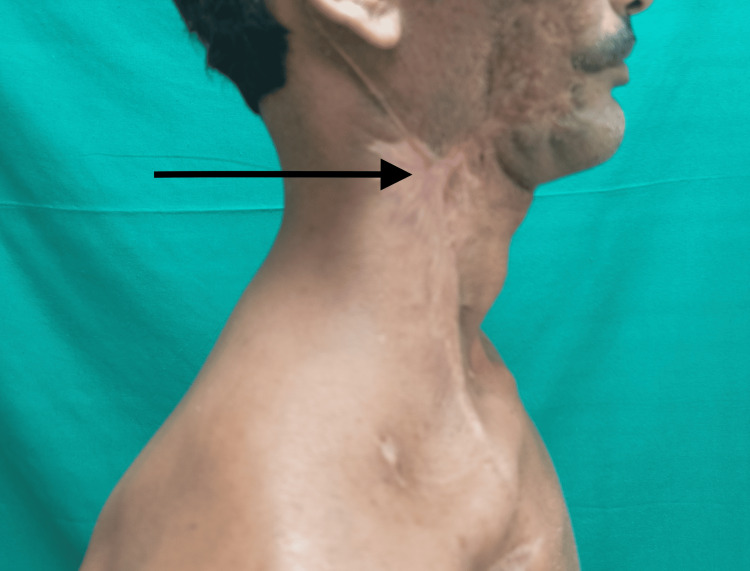
Follow-up picture at three months postoperatively with the healthy wound (black arrow)

From POD 11th-21st, healthy granulation tissue appeared. The patient was started on oral fluids as there was no fistula from the oral cavity. The intraoral paddle showing the skin is shown in Figure 5. The wound was subsequently managed with local wound care and secondary suturing (Figure 6).

## Discussion

Free flap failure occurs mostly due to thrombosed anastomosis, usually within the first 48 hours [3]. The salvage rate of late anastomotic thrombosis (on or after the fifth POD) has been reported to be 60.8% when addressed with immediate surgical intervention [2]. Early thrombotic events (<48 hours) are usually salvaged by simple revision of MVA. However, surgical revision is usually insufficient to resolve vascular insufficiency of late-onset (>48 hours) [4,5]. Delayed partial flap compromise is a rare and perplexing problem. The quandary is whether to re-explore to check for patency of anastomosis or watchfully wait. 

The problems with re-exploration in such cases are densely fibrosed and inflamed neck field [6]. Identification of structures is difficult and inadvertent injuries to flap or donor vessels are possible. De-insetting the flap for re-exploration disrupts the neovascularization from the wound edges and carries the risk of the devascularization of the skin paddle [5]. In this case, we decided to watchfully wait and observe. While the congestion initially progressed, there were areas of normal vascularity in the flap. Eventually, there was a clear demarcation between viable and non-viable areas, which were debrided. The exact cause of flap congestion could not be ascertained but there was suspected partial disruption of the venous anastomosis during violent neck movement leading to gradual thrombosis or compression from the tracheostomy tube over the pedicle running across the midline.

Assuming that the vascular pedicle was compromised, one can attribute the survival of the flap to neovascularization, which can occur as early as the 4th POD, decreasing the sole dependence of the flap on the pedicle vessels [5,7]. Therefore, in the late postoperative period, the decision about surgical revision is more critical.

The likely explanation of flap behavior in our case is that there was a partial venous thrombosis in the perforator vein or MVA between the flap vein and vein graft or thrombosis of some arborizing branches inside the flap after piercing the deep fascia. The inner mucosal part of the ALT flap survived due to the escape of some branch to any thrombosis or due to neovascularization from the inset edges. The outer flap also partially survived due to neovascularization. 

In the current case with delayed partial congestion of the flap, we adopted the strategy of watchful waiting. While the strategy of masterly inactivity is known, the evidence is mostly anecdotal. There is a relative paucity of literature regarding the strategies to adopt when faced with similar situations. Hence, we feel it is necessary to report the approach we followed in this particular case. This report can contribute to the formulation of guidelines for the management of flap complications.

## Conclusions

This case report shows that watchful waiting can be a successful strategy for flap salvage when confronted with delayed, partially compromised free flaps, especially when salvage/lifeboat options are already exhausted. While the strategy of masterly inactivity is known the evidence is mostly anecdotal, bearing in mind the risks of late revision surgery and the reported compromised results. We suggest considering a watch-and-wait approach especially when viable areas persist and the flap congestion is not generalized. Hence, we feel it is necessary to report the approach followed in this case.
